#  Prevalence and Diversity of Leptospires in Different Ecological Niches of Urban and Rural Areas of South Andaman Island

**DOI:** 10.1264/jsme2.ME15149

**Published:** 2016-03-03

**Authors:** Chandan Lall, K. Vinod Kumar, R. Vimal Raj, K. Vedhagiri, P. Vijayachari

**Affiliations:** 1 Regional Medical Research Centre (ICMR), WHO Collaborating Centre for Diagnosis, Reference, Research and Training in LeptospirosisPort Blair-744101, Andaman and Nicobar IslandsIndia; 2 National Hub for Healthcare Instrumentation Development (NHHID), Centre for Biotechnology, Anna UniversityChennai—600 025India

**Keywords:** *Leptospira*, ecology, intermediate, diversity, zoonotic, environment

## Abstract

Leptospirosis is an emerging disease around the globe. South Andaman Island is an endemic region for leptospirosis. We herein compared the prevalence of leptospires in urban and rural areas of South Andaman Island. The PCR detection and isolation of *Leptospira* revealed that pathogenic leptospires were prevalent in sewage water and household drainage water in urban areas and in paddy fields, vegetable field water, and stream water in rural areas. These results demonstrate that intermediates are ubiquitously present in the environment and may be responsible for asymptomatic infections, and also provide an insight into disease ecology.

Leptospirosis is a widely spread zoonotic disease that has a significant health impact in many parts of the world and primarily affects vulnerable populations (20). The causative agent of the disease, *Leptospira* is acquired by humans indirectly from contaminated environmental surface waters or direct contact with the contaminated urine of domestic and wild mammals (5). The genus *Leptospira* contains pathogens that have been serologically classified into more than 250 serovars, intermediate pathogens, and saprophytes with genetic classifications into 21 different species (17). Leptospirosis is highly endemic in The Andaman Islands, with the majority of the population being exposed to the disease for more than the last eight decades (13, 16, 19). Agriculture laborers, forest workers, sewage workers, animal handlers, and butchers are in the high-risk group. Members of the population at risk acquire this infection from either the environment or animals (14). Serological studies detected anti-leptospiral antibodies in 55% of the healthy population in the North Andaman, Andaman, and Nicobar archipelago (12). High seroprevalence among a healthy population in a hyperendemic area may be the result of asymptomatic infection (2, 6). *Leptospira* has been detected in soil and water samples from various sources and in different geographical areas in rural and urban settings in some countries (4, 9, 11). Since the presence of *Leptospira* and its diversity in the environment have been suggested to play a role in the transmission dynamics of the disease, this study was designed to reveal the presence of various species of *Leptospira* in different types of water in urban and rural parts of South Andaman Island.

The Andaman and Nicobar Archipelago is situated between 6°N and 14°N latitude and 92°E and 94°E longitude in the Bay of Bengal and is spread over a linear distance of more than 550 km and geographical area of 8,249 km^2^. The present study was conducted in rural and urban settings of South Andaman Island.

A total of 246 water samples were collected from different sampling sites in urban and rural settings of South Andaman Island between June 2012 and November 2012, as described by Henry and Johnson (8), with minor modifications. Approximately 20 mL of water was centrifuged at 8,000×*g* for 10 min. DNA was extracted from the pellet using a commercial kit QIAamp DNA Mini Kit (Qiagen, Valencia, California, USA) as per the manufacturer’s instructions. DNA samples were processed to detect the presence of any (saprophytic, intermediate, and pathogenic leptospires) leptospiral DNA by PCR using G1&G2 and B64I & B64II primers (7, 10). Pathogenic leptospires were identified using Lipl 32 primers that detect the Lipl32 gene, which encodes an outer membrane protein specifically present in pathogens (1).

Approximately 50-mL water samples were filtered through a sterile membrane filter with a pore size of 0.22 μm, and 0.5 mL of the samples was inoculated in duplicate into 4.5-mL semi-solid Ellinghausen-McCullough-Johnson-Harris (EMJH) medium containing 5-fluorouracil (5-FU) at a concentration of 1 mg mL^−1^. These tubes were incubated at 30°C and checked daily by dark-field microscopy for the presence of *Leptospira*. Samples were considered negative if no leptospires were detected after a 28-d incubation. Pure cultures were obtained by single-colony isolation using solid LVW agar medium. LVW agar medium was inoculated with a diluted liquid culture of bacteria (10^4^ cells mL^−1^) and incubated at 30°C for 28 days. A single colony was inoculated into liquid EMJH medium and incubated at 30°C.

Regarding DNA extraction, a confluent culture of isolates was harvested by centrifugation (16,000×*g* for 3 min) at 4°C. DNA was extracted from the pellet using a commercial QIAamp DNA Mini Kit (Qiagen) as per the manufacturer’s instructions. The gene sequence from the extracted DNA was amplified using 16S rRNA primers as described earlier (1). Amplified DNA products from representative isolates were verified by DNA sequencing. The sequences of the other *Leptospira* species used for alignment and to calculate levels of homology were obtained from GenBank. A phylogenetic tree was constructed using the neighbor-joining method by MEGA version 6.06.

Out of 113 samples collected from urban areas, 11 (9.7%) were positive for pathogenic and 50 (44.2%) were positive for other leptospiral DNA (intermediately pathogenic or saprophytic leptospires). With the highest positivity, 5 (9.8%) sewage water samples were positive for pathogenic leptospires, while 28 (54.9%) sewage water samples were positive for other leptospiral DNA. Six (18.7%) household drainage water samples were positive for pathogenic leptospires, while 14 (43.7%) samples were positive for other leptospiral DNA. Similarly, out of 133 samples collected from rural areas, 9 (6.7%) were positive for pathogenic leptospires, while 58 (43.6%) were positive for other leptospires. One (6.25%) of the vegetable field samples was positive for pathogenic leptospires, while 7 (46.6%) were positive for the presence of other leptospiral DNA. Seven (15.9%) of the paddy field water samples were positive for pathogenic leptospires, while 19 (43.18%) were positive for other leptospiral DNA. One (5%) of the stream water samples was positive for pathogenic leptospiral DNA, whereas 10 (50%) were positive for leptospires (Table 1).

Forty-nine isolates were recovered from the samples collected, 16 of which were isolated from urban area samples, 6 from sewage water, 7 from household drainage water, and 3 from market area drainage water. Thirty-three isolates were recovered from rural area samples. Of these, 19 isolates were recovered from paddy field water, 6 from vegetable field water, 3 from forest land water, 3 from cow shed drainage, 1 from household drainage, and 1 from pond water. The present study showed that 83% of the isolates were intermediately pathogenic. *L. wolffii* was ubiquitously present in urban and rural environments, except for pond water and civic toilet drainage. None of the isolates were recovered from slaughter house drainage water, pond water (urban), or streams, which may have been due to the heavy contamination of other bacteria and toxic chemicals in these samples. The isolation of saprophytic *L. meyeri* (ELI 48 RMRC, ELI 49 RMRC) and pathogenic *L. interrogans* (ELI 3-RMRC) from urban household drainage indicates the maintenance of poor sanitation in the surroundings. *L. licerasiae* was only found in samples collected from rural parts of South Andaman in animal house drainage, paddy field water, vegetable field water, and household drainage.

Since intermediately pathogenic leptospires are known to have the potential to cause human and animal leptospirosis (3, 21), asymptomatic infections and mild cases of leptospirosis, which are generally under reported, may be due to the abundance of intermediately pathogenic leptospires in the environment (6). Diverse species of *Leptospira* were isolated from paddy field water samples and vegetable field water samples including *L. interrogans*, *L. licerasiae*, and *L. wolffii*, as shown in Fig. 1. In addition to these isolates, ELI 45-RMRC and ELI-31-RMRC, which belong to *L. kmeyti*, were isolated from paddy field samples only. Based on the results of the present study, it is evident that the paddy field environment supports diverse species of *Leptospira* and this may be related to the high seroprevalence in agriculture workers (15, 18) (Fig. 2). However, the importance of the circulation of intermediately pathogenic leptospires in the ecology of the disease remains to be studied. The determination of pathogenic leptospires in the environmental in rural and urban settings of South Andaman Island emphasizes the need for appropriate sanitary measures, the proper disposal of waste, and use of protective gear to reduce exposure.

## GenBank accession numbers of the sequences of isolates

KT804578, KT804579, KT804580, KT804581, KT804582, KT804583, KT804584, KT804585, KT804586, KT804587, KT804588, KT804589, KT804590, KT804591, KT804592, KT804593, KT804594, KT804595, KT804596, KT804597, KT804598, KT804599, KT804600, KT804601, KT804602, KT804603, KT804604, KT804605, KT804606, KT804607, KT804608, KT804609, KT804610, KT804611, KT804612, KT804613, KT804614, KT804615, KT804616, KT804617, KT804618, KT804619, KT804620, KT804621, KT804622, KT804623, KT804624, KT804625, KT804626,

## Figures and Tables

**Fig. 1:**
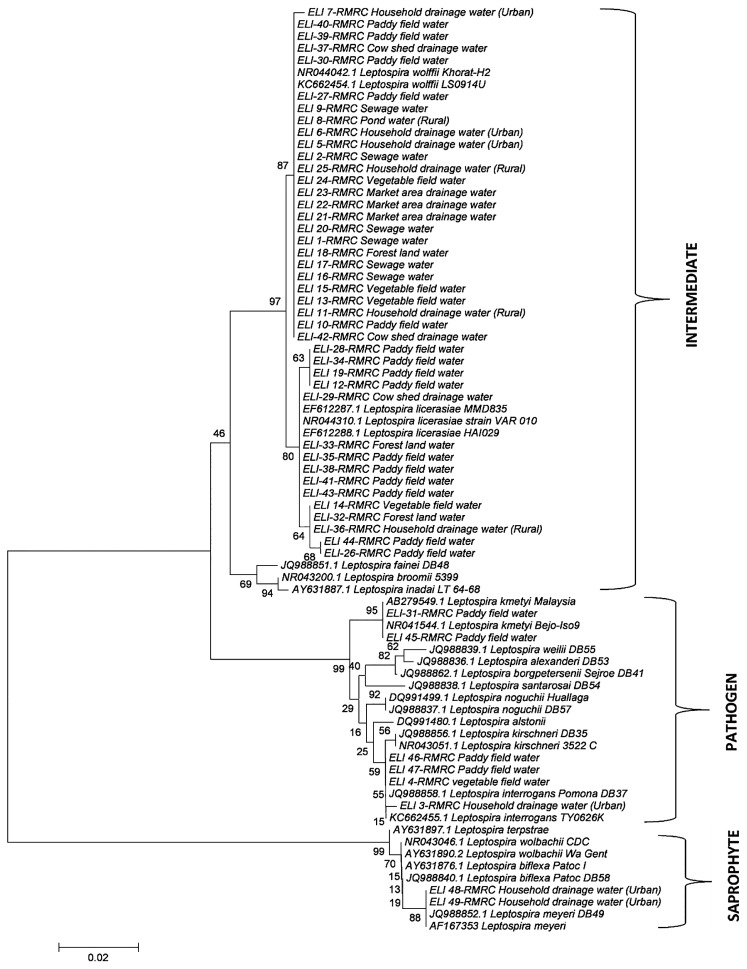
Phylogenetic tree analysis of 16S rRNA sequences of *Leptospira* isolated from environmental water samples.

**Fig. 2:**
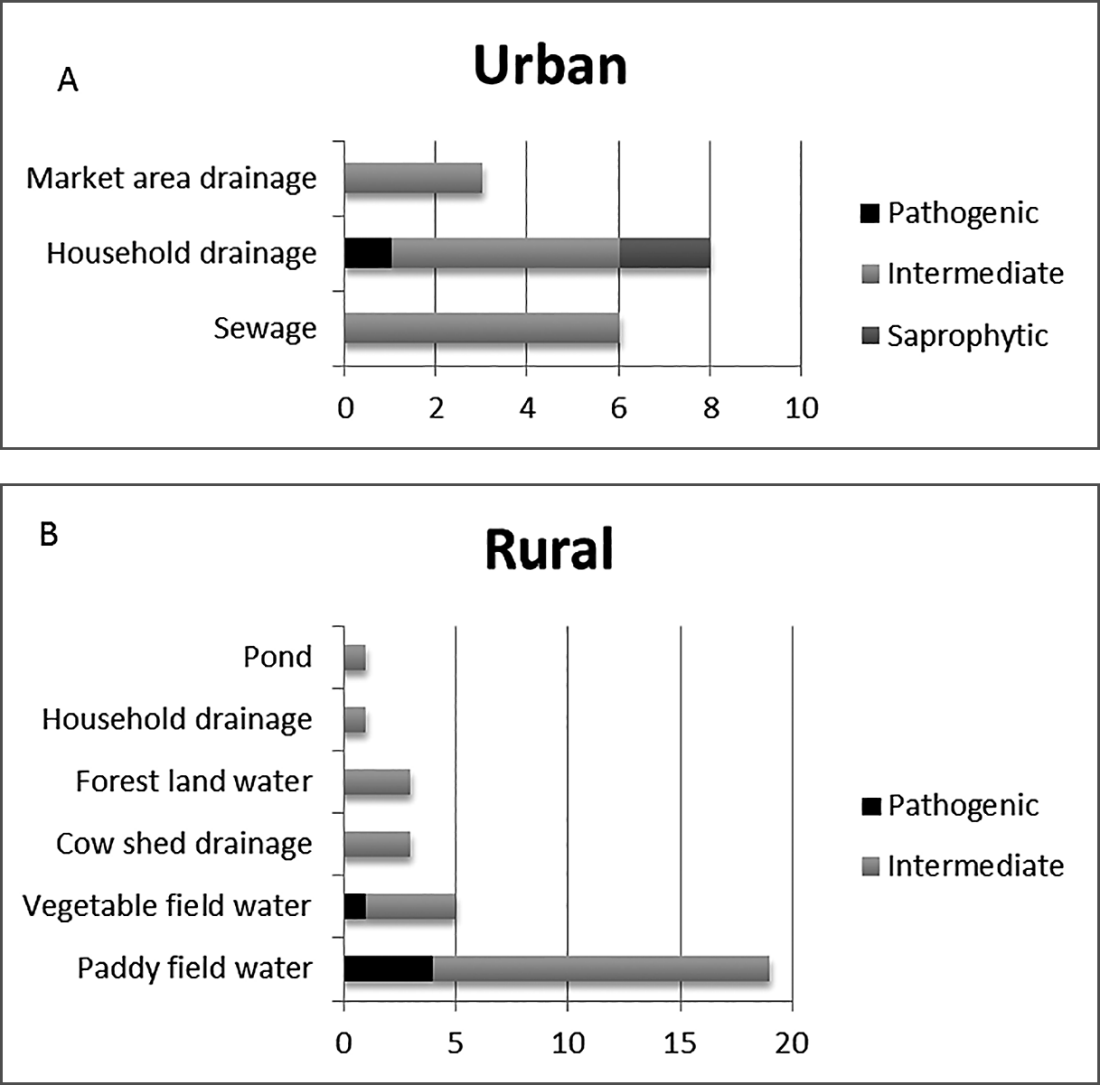
A and B Distribution of Pathogenic, Intermediately Pathogenic, and Saprophytic *Leptospira* in urban and rural settings of South Andaman Island.

**Table 1:** Distribution of *Leptospira* PCR-positive samples in rural and urban areas of South Andaman Island.

Area	Sources of water	Number of samples	Pathogenic leptospires	Other leptospires
Urban				
	Sewage	51	5 (9.8%)	28 (54.9%)
	Household drainage	32	6 (18.7%)	14 (43.7%)
	Market area drainage	7	0 (0%)	4 (57.1%)
	Slaughter house drainage	9	0 (0%)	4 (44.4%)
	Civic toilet drainage	10	0 (0%)	0 (0%)
	Pond	4	0 (0%)	0 (0%)

	Total	113	11 (9.7%)	50 (44.2%)

Rural				
	Paddy field water	44	7 (15.9%)	19 (43.1%)
	Stream	20	1 (5%)	10 (50%)
	Household drainage	12	0 (0%)	4 (33.3%)
	Cowshed drainage	8	0 (0%)	4 (50%)
	Pond	8	0 (0%)	2 (25%)
	Forest land water	26	0 (0%)	12 (46.1%)
	Vegetable field water	15	1 (6.6%)	7 (46.6%)

	Total	133	9 (6.7%)	58 (43.6%)
